# Supplementary material for: Boosting Photodetection via Plasmonic Coupling in Quasi‐2D Mixed‐n Ruddlesden‐Popper Perovskite Nanostripes
Source: Small. 2025 Nov 30;22(5):e09443. doi: 10.1002/smll.202509443 (PMC12824564; doi:10.1002/smll.202509443)
Supplement: Supplementary file 1 — Supporting Information [file SMLL-22-e09443-s001.docx]

**Supporting Information**

Boosting Photodetection via Plasmonic Coupling in Quasi-2D Mixed-n Ruddlesden-Popper Perovskite Nanostripes

Brindhu Malani S,*^,a^ Eugen Klein,^a^ Ronja Maria Piehler,^a^ Rostyslav Lesyuk ^ab^ and Christian Klinke*^,acd^

^a^ *Institute of Physics, University of Rostock, Albert-Einstein-Straße 23, 18059 Rostock, Germany*

^b^ *Pidstryhach Institute for Applied Problems of Mechanics and Mathematics of NAS of Ukraine, Naukowa Str. 3b, 79060 Lviv, Ukraine*

^c^ *Department Life, Light & Matter, University of Rostock, Albert-Einstein-Strasse 25, 18059 Rostock, Germany*

^d^ *Department of Chemistry, Swansea University – Singleton Park, Swansea SA2 8PP, United Kingdom*

^*^ *Corresponding Authors:* [*brindhu.seelan@uni-rostock.de*](mailto:brindhu.seelan@uni-rostock.de)*,* [*christian.klinke@uni–rostock.de*](mailto:Christian.klinke@uni–rostock.de)


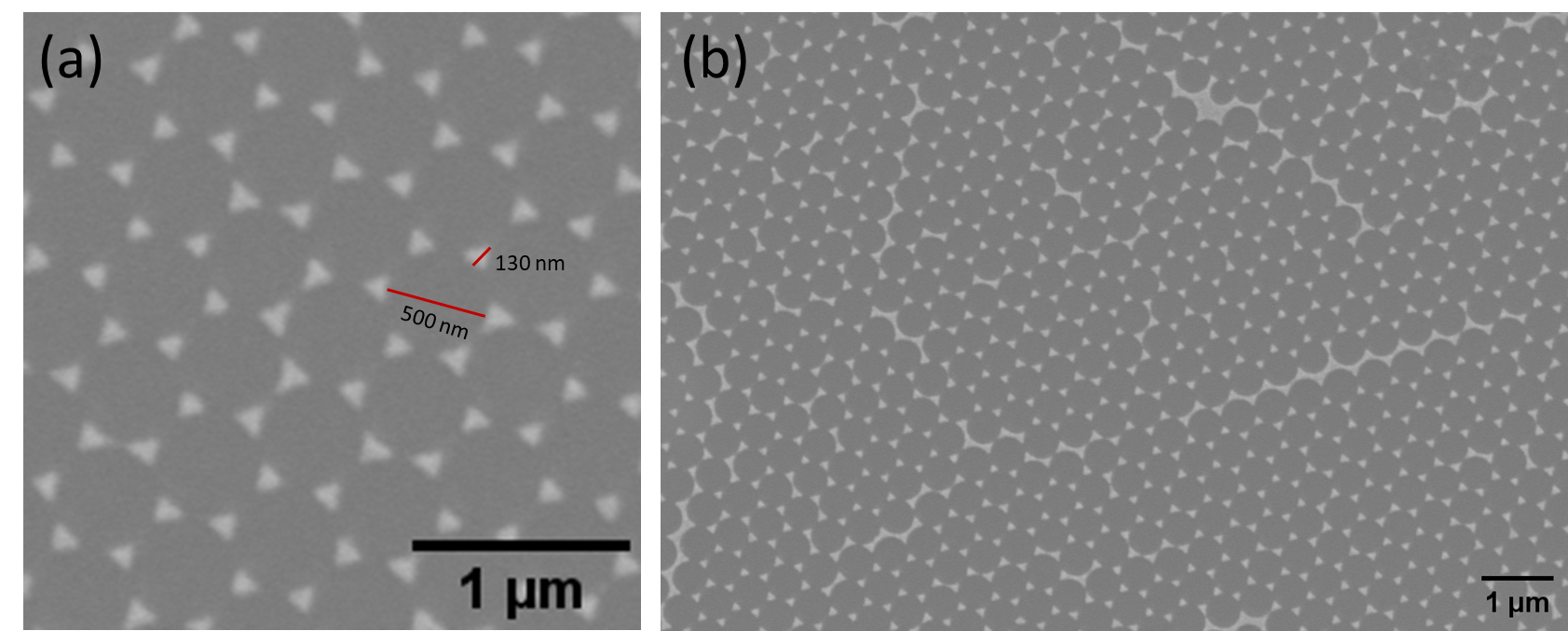


**Figure S1.** SEM images of Ag nanostructure arrays (ANA) on SiO_2_/Si substrate fabricated using colloidal lithography.


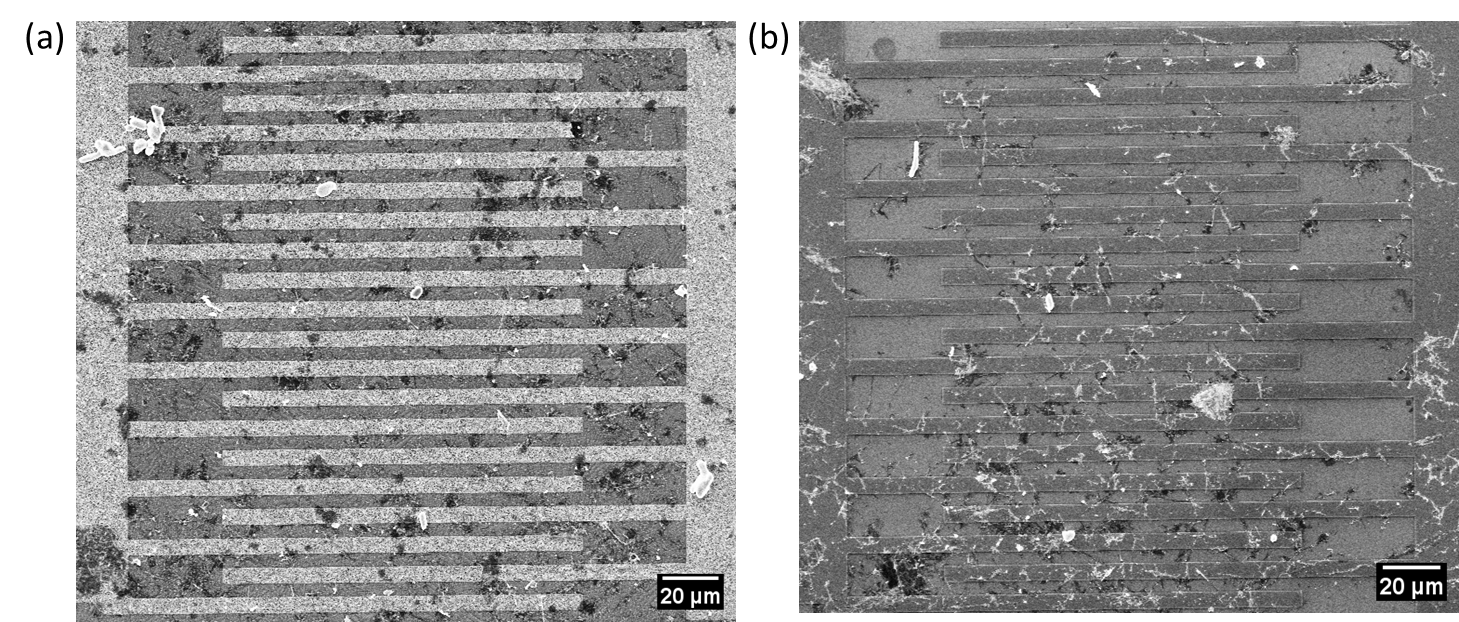


**Figure S2.** SEM images of (a) plasmonic-perovskite hybrid and (b) reference photodetector. The nanostripes between the electrode spacing are calculated to estimate the active area.

|  | **Absorbance**  **(nm [eV])** | **PL**  **(nm [eV])** | **Layer number**  **(*n* =)** |
| --- | --- | --- | --- |
| Nanostripes | 417 [2.97] | 420 [2.95] | 2 |
|  | 449 [2.76] | 455 [2.72] | 5 |
|  | 468 [2.65] | 470 [2.64] | 7 |
|  | 483 [2.57] | 485 [2.56] | 8 |
|  | 523 [2.37] | 524 [2.36] | bulk |

**Table S1.** The absorbance and PL spectral features have been assigned to the corresponding layer numbers based on previous studies. ^[1–3]^


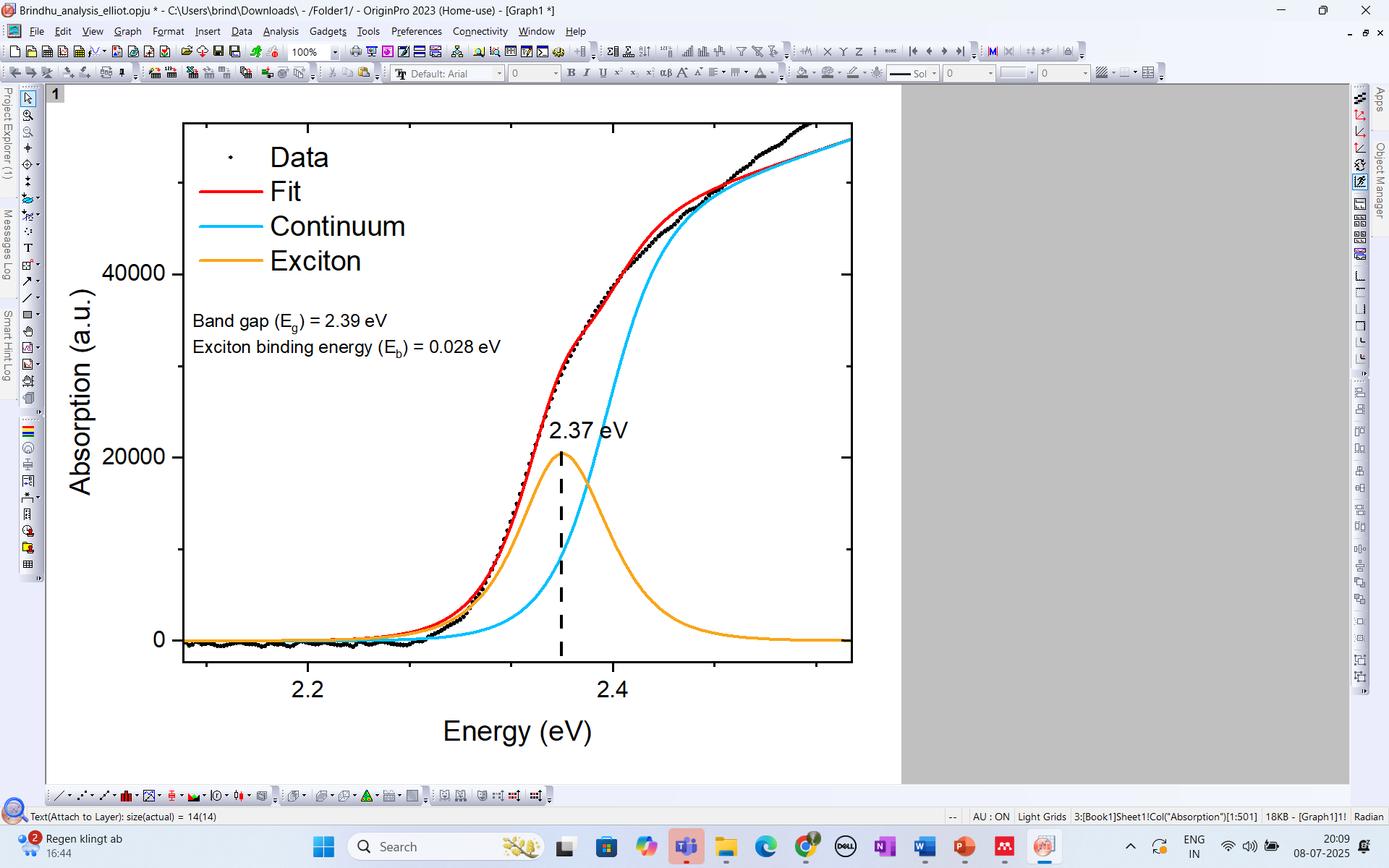


**Figure S3.** Elliott model fitting to determine the exciton peak position.


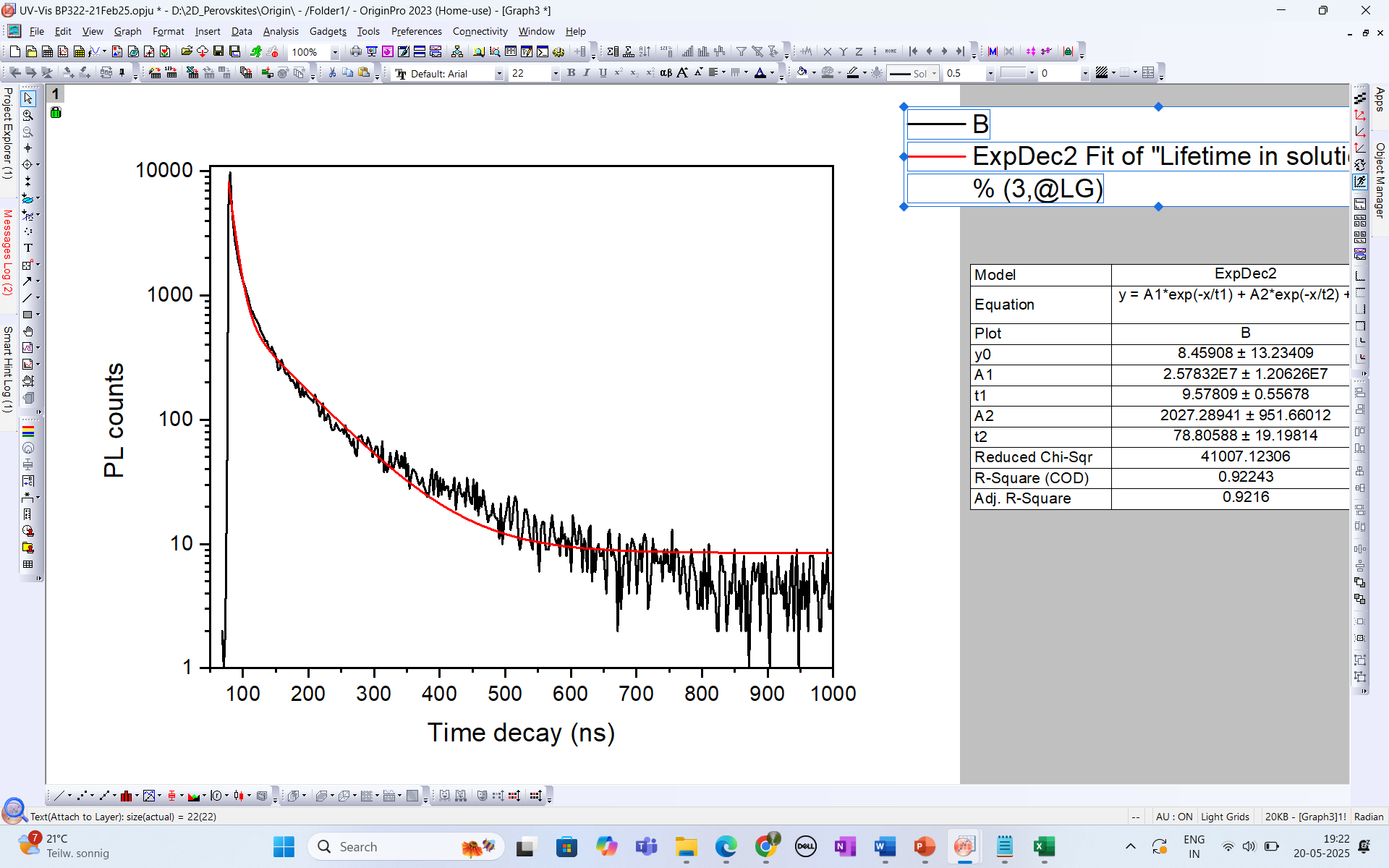


**Figure S4.** TRPL profile for perovskite nanostripes in solution.

|  | **τ_1_**  **(ns)** | **A_1_** | **τ_2_**  **(ns)** | **A_2_** | **<τ>**  **(ns)** | **<rate>**  **(1/ns)** |
| --- | --- | --- | --- | --- | --- | --- |
| Solution | 9.57 | 2.57E+7 | 78.80 | 2027 | 9.61 | 0.10 |
| SiO_2_/Si | 0.77 | 543 | 6.50 | 63 | 3.61 | 0.27 |
| ODT/ANA | 0.48 | 4026 | 3.28 | 38 | 0.64 | 1.54 |
| ANA | 0.82 | 607 | 4.60 | 61 | 2.18 | 0.45 |

**Table S2.** Fitting parameters for TRPL profiles presented in Figure S4 and Figure 3 (d).


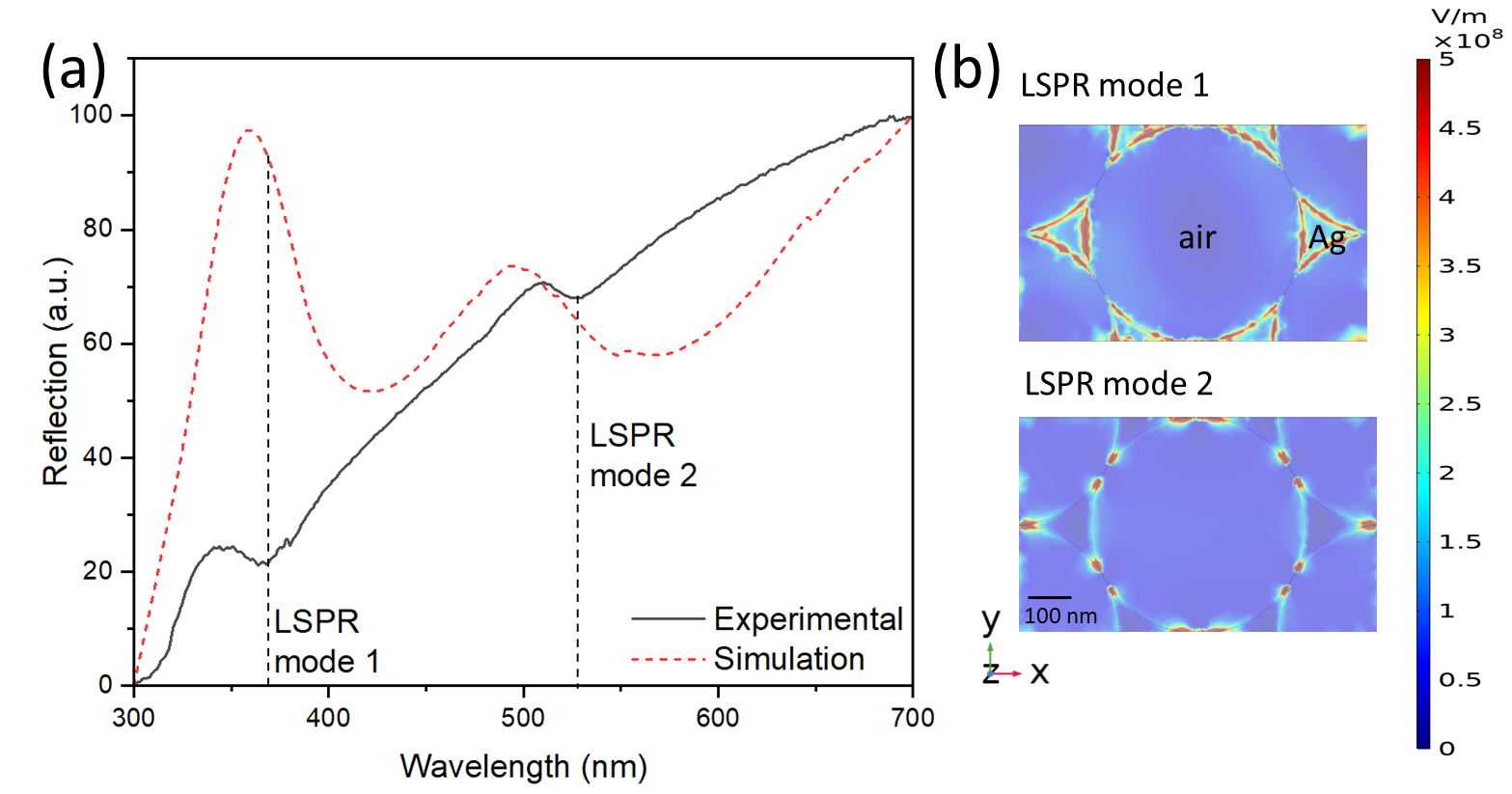


**Figure S5.** Simulated (a) reflection curves for ANA. (b) electric field distribution (V/m) for dip occurring at around LSPR modes 1 and 2.


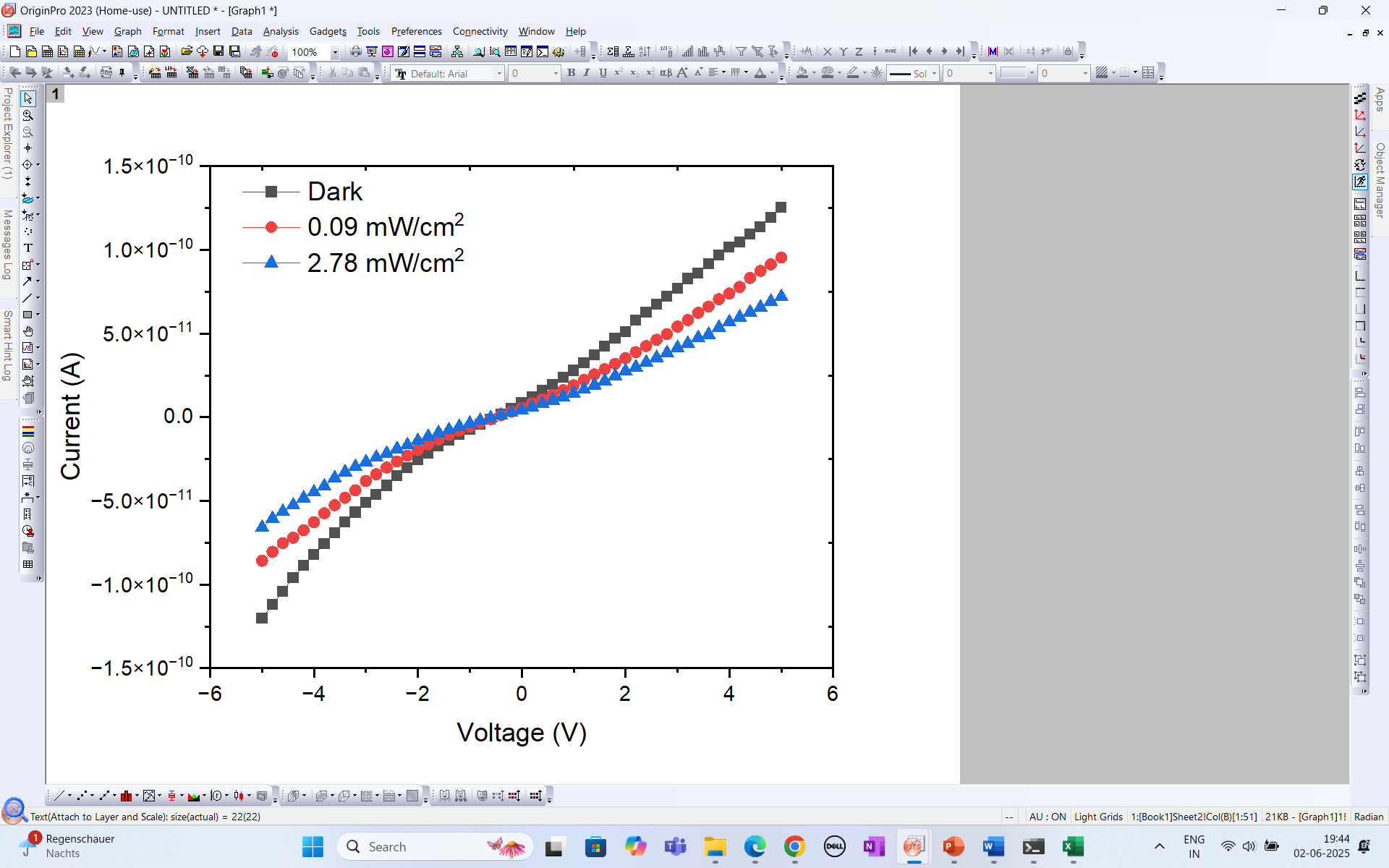


**Figure S6.** Current-voltage (I-V) measurements for ANA substrate show a decrease in photocurrent with light illumination.


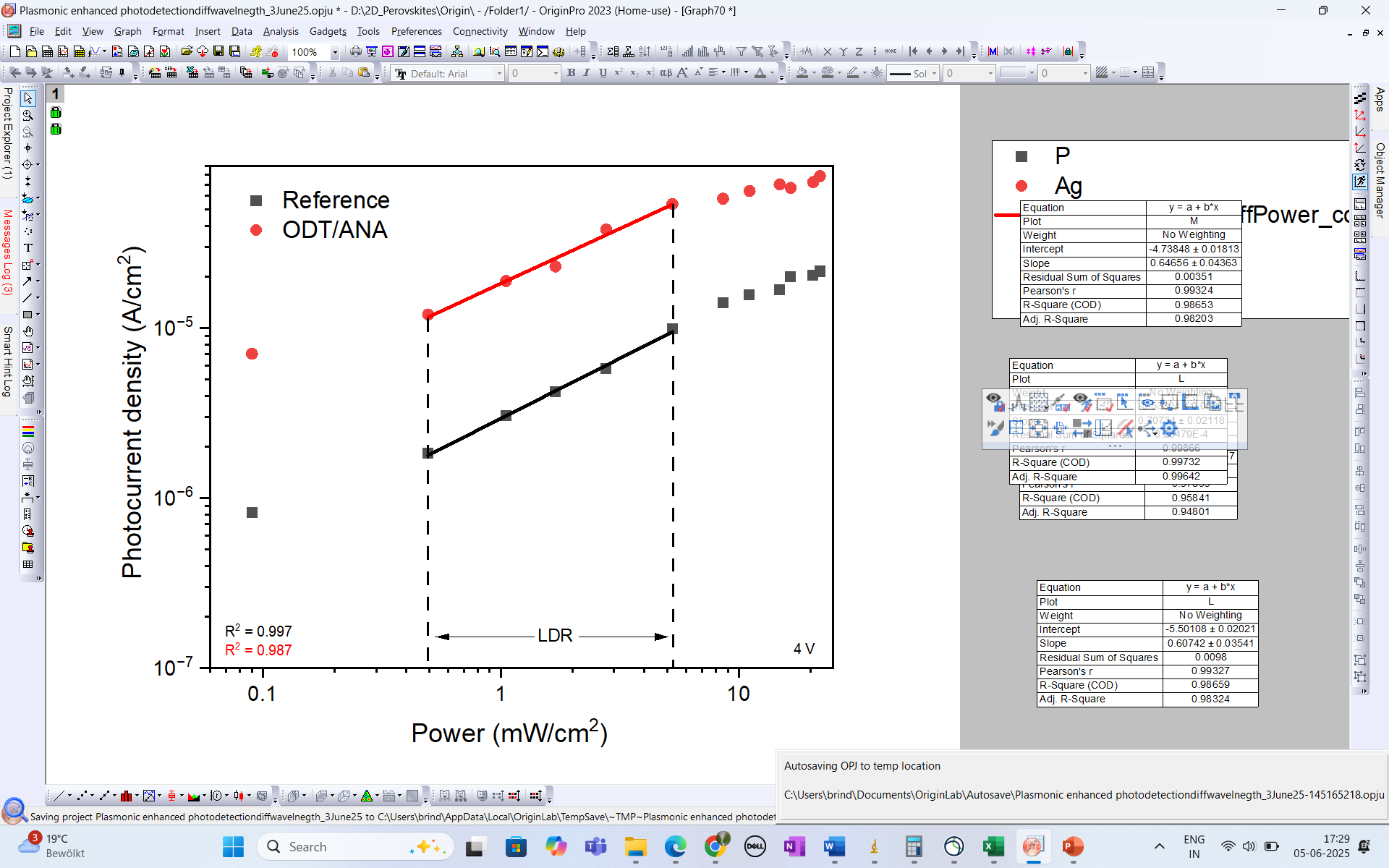


**Figure S7.** Photocurrent density measured at different illumination power at a bias voltage of 4 V displaying linear dynamic range (LDR).


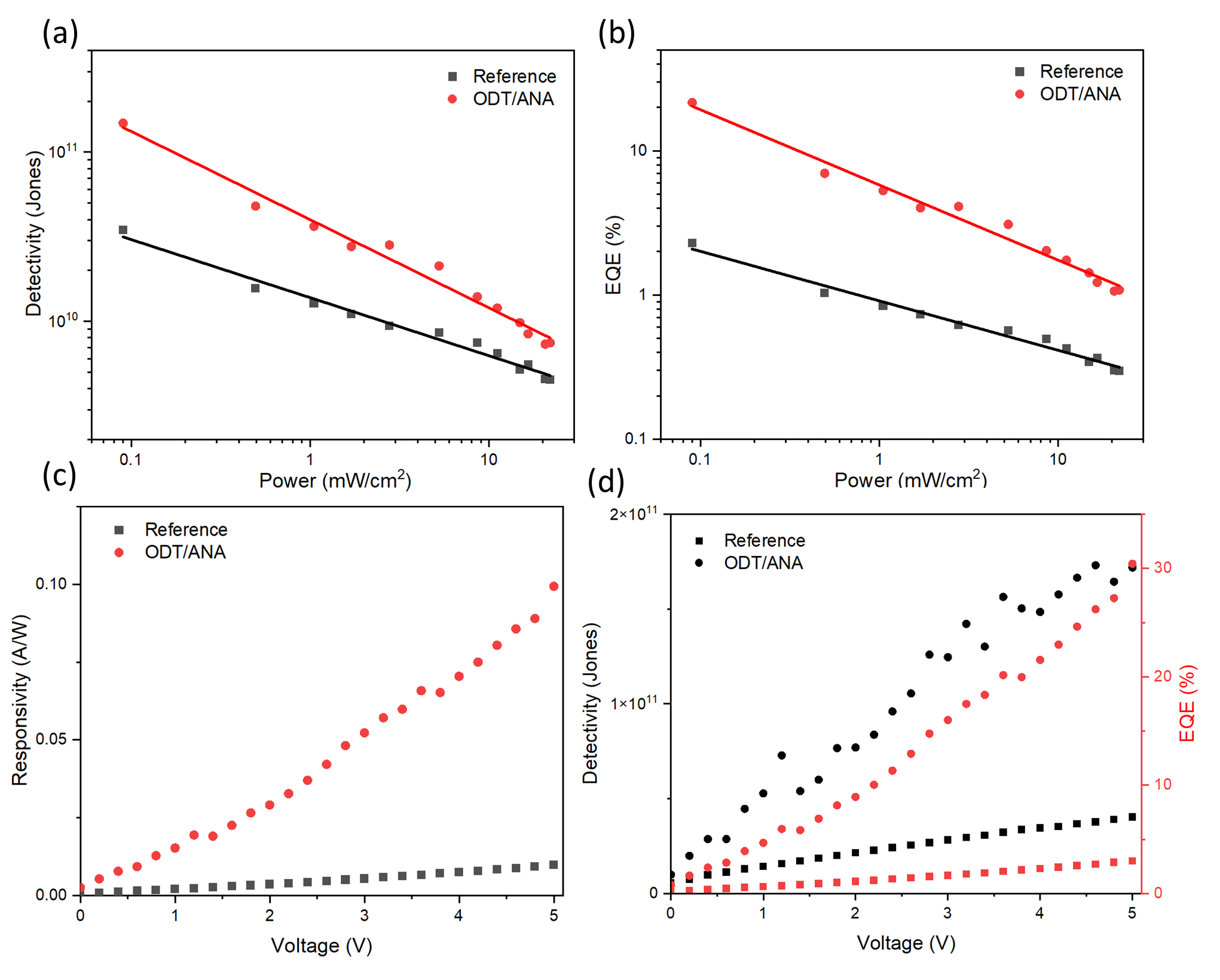


**Figure S8.** Photodetection performance of reference and ODT/ANA PDs (a) Detectivity (b) EQE at different illumination power at a bias voltage of 4 V. (c) Responsivity (d) Detectivity and EQE at a power density of 0.09 mW/cm^2^.


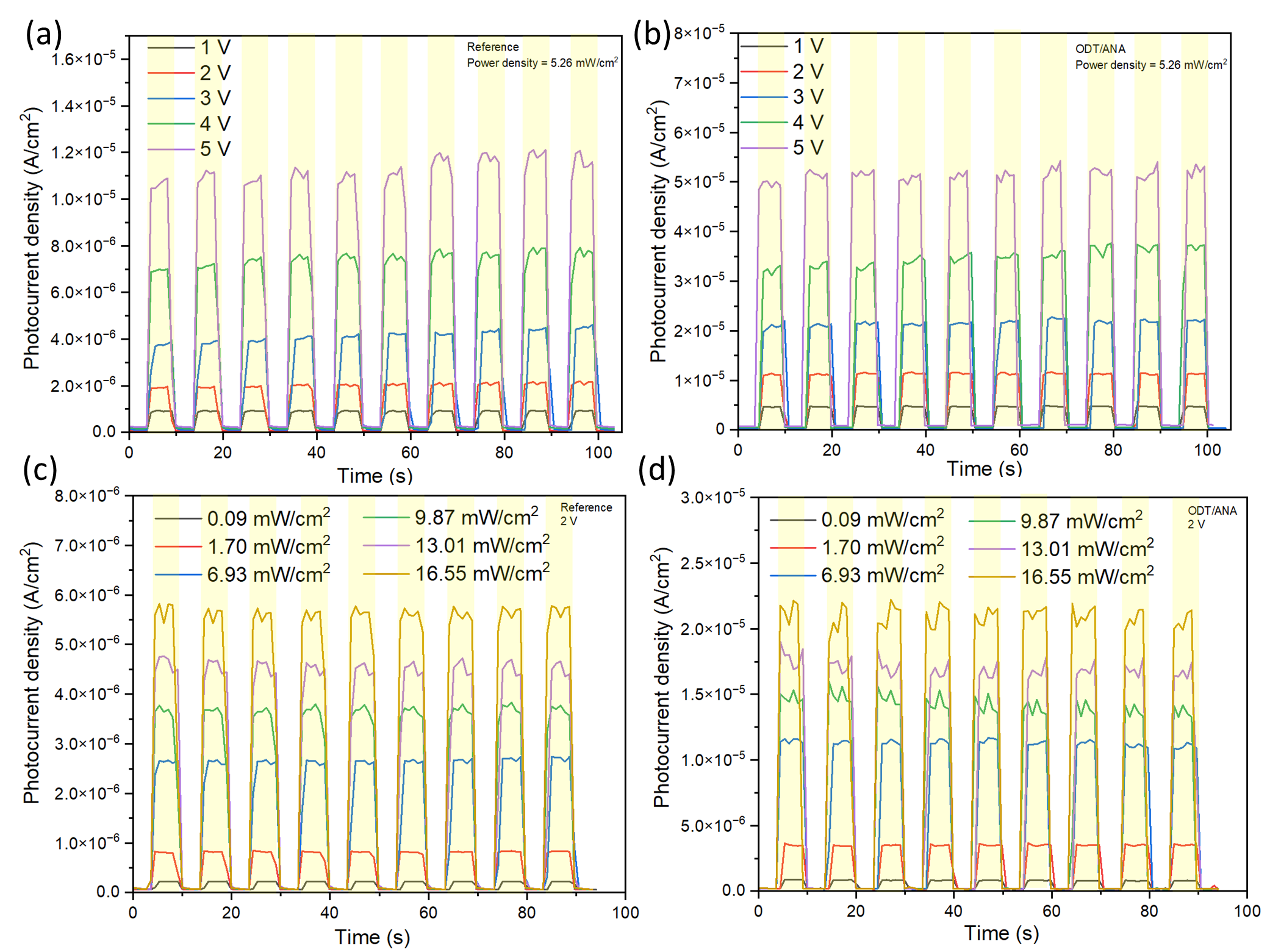


**Figure S9.** Transient photocurrent measurements for (a, c) reference and (b, d) ODT/ANA PDs at (a, b) different bias voltages at power 5.2 mW/cm^2^ and (c, d) illumination power at 2 V bias using an optical signal with a period of 10 s.


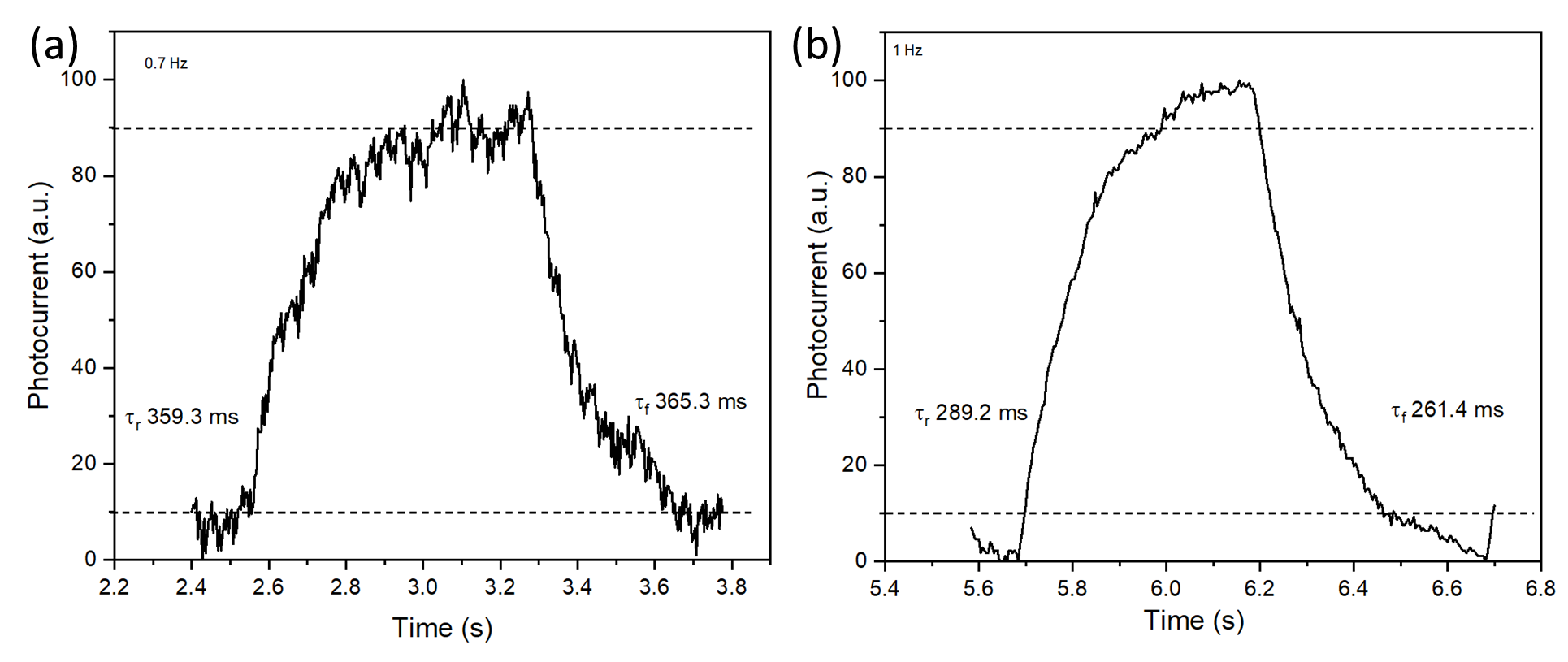


**Figure S10.** Temporal response for (a) reference and (b) ODT/ANA PD at around 3dB frequency.

**Reference**

[1] E. Klein, A. Black, Ö. Tokmak, C. Strelow, R. Lesyuk, C. Klinke, Micron-Size Two-Dimensional Methylammonium Lead Halide Perovskites. *ACS Nano* **2019**, *13 (6)*, 6955–6962. DOI 10.1021/acsnano.9b01907.

[2] M. Rahil, R. M. Ansari, S. Ahmad, S. S. Islam, Nanostructured Ruddlesden–Popper-Layered Lead Bromide Perovskites with Stable and Selected Wavelength for Photodetection Applications. *ACS Appl Nano Mater* **2023**, *6 (7)*, 5187–5199. DOI 10.1021/acsanm.2c05092.

[3] A. Niebur, E. Klein, R. Lesyuk, C. Klinke, J. Lauth, Understanding the Optoelectronic Processes in Colloidal 2D Multi‐Layered MAPbBr_3_ Perovskite Nanosheets: Funneling, Recombination and Self‐Trapped Excitons. *Adv Opt Mater* **2025**, *13 (12)*, DOI 10.1002/adom.202402923.
